# *Scoredist*: A simple and robust protein sequence distance estimator

**DOI:** 10.1186/1471-2105-6-108

**Published:** 2005-04-27

**Authors:** Erik LL Sonnhammer, Volker Hollich

**Affiliations:** 1 Center for Genomics and Bioinformatics, Karolinska Institutet, Berzelius väg 35, 171 77 Stockholm, Sweden

## Abstract

**Background:**

Distance-based methods are popular for reconstructing evolutionary trees thanks to their speed and generality. A number of methods exist for estimating distances from sequence alignments, which often involves some sort of correction for multiple substitutions. The problem is to accurately estimate the number of true substitutions given an observed alignment. So far, the most accurate protein distance estimators have looked for the optimal matrix in a series of transition probability matrices, *e.g. *the Dayhoff series. The evolutionary distance between two aligned sequences is here estimated as the evolutionary distance of the optimal matrix. The optimal matrix can be found either by an iterative search for the Maximum Likelihood matrix, or by integration to find the Expected Distance. As a consequence, these methods are more complex to implement and computationally heavier than correction-based methods. Another problem is that the result may vary substantially depending on the evolutionary model used for the matrices. An ideal distance estimator should produce consistent and accurate distances independent of the evolutionary model used.

**Results:**

We propose a correction-based protein sequence estimator called *Scoredist*. It uses a logarithmic correction of observed divergence based on the alignment score according to the BLOSUM62 score matrix. We evaluated *Scoredist *and a number of optimal matrix methods using three evolutionary models for both training and testing Dayhoff, Jones-Taylor-Thornton, and Müller-Vingron, as well as Whelan and Goldman solely for testing. Test alignments with known distances between 0.01 and 2 substitutions per position (1–200 PAM) were simulated using ROSE. *Scoredist *proved as accurate as the optimal matrix methods, yet substantially more robust. When trained on one model but tested on another one, *Scoredist *was nearly always more accurate. The Jukes-Cantor and Kimura correction methods were also tested, but were substantially less accurate.

**Conclusion:**

The *Scoredist *distance estimator is fast to implement and run, and combines robustness with accuracy. *Scoredist *has been incorporated into the Belvu alignment viewer, which is available at .

## Background

Estimating divergence time of protein sequences is one of the fundamental problems in bioinformatics. Evolutionary distance estimates are used by many of the most commonly used phylogenetic tree reconstruction algorithms [1-3]. In current research, phylogenetic trees are used for many types of subsequent analysis, *e.g. *orthology inference [4-6]. Early models for sequence evolution focussed on nucleotides. They commonly employ Markov chains and assume independent evolution at every site. Each of the four nucleotides is identified by one state and the substitution probability is modelled as a state transition probability from one state to another. In the most straightforward approach, the same state transition probability is assigned to every substitution [7]. Subsequent models take account of more nucleotide specific properties, *e.g. *transitional and transversional substitutions as well as GC content (see [8] for an introduction). These more advanced approaches are bound to nucleotide sequences and cannot be directly used with protein sequences.

Markov chain models for protein evolution differ from nucleotide approaches in their larger number of states and transitions for which parameters need to be estimated. The protein sequence Jukes-Cantor model assigns the same probability to each substitution and is hence a rather poor approximation. This method essentially takes the observed differences between two sequences and corrects this value to the estimated evolutionary distance using a logarithmic function. Other similar methods exist that also correct observed differences, *e.g. *Kimura's method [9]. Although they produce rather inaccurate distance estimates, correction-based distance estimators are popular because of their simplicity. More advanced protein evolution models estimate parameters from protein sequence alignments. Assuming the same substitutions for closely and distantly related sequences leads to the construction of the Dayhoff matrix series [10]. Following this approach, it suffices to collect data from alignments of closely related sequences to build an evolutionary model of amino acid substitution.

Dayhoff and co-workers introduced the term Percent Accepted (point) Mutation (PAM), which denotes a commonly used measure for evolutionary distance between two aligned sequences (insertions and deletions are ignored). In other words, two sequences at a distance of 150 PAM are related to each other by 1.5 substitutions per position on average. As substitution is a stochastic process, some positions will experience multiple substitutions while others will experience none. It is also possible that secondary substitutions at one site will result in the original residue, making the evolutionary steps invisible. This is in essence the reason why estimating evolutionary distance is so hard – multiple substitutions cannot be observed directly. An evolutionary distance of 250 PAM corresponds roughly to 80% observed differences. The term PAM is found in literature for both the matrix series given by Dayhoff et al. as well as for evolutionary distance unit. In this publication we refer to the matrices as Dayhoff matrices and reserve the term PAM for distance units.

There are two major shortcomings connected with the derivation of the Dayhoff matrices. First, potential errors inherent in the experimental data will be magnified by extrapolation. Additionally, it is questionable whether substitution probabilities observed on closely related sequences can accurately reflect the evolution of more distantly related sequences. The efforts of researchers since the publication of the Dayhoff matrices have led to several other matrix series, sharing the idea of an underlying Markov chain. They differ in terms of the data they are built upon and account for the above-mentioned shortcomings in various ways [11-13].

The approach behind the BLOSUM matrices [14] is different from Dayhoff's evolutionary model. Whereas the Markov model assumes that any transition probability matrix may be derived from another matrix in the same series, the BLOSUM matrices do not imply any evolutionary time. There is no direct mathematical relationship between matrices in the BLOSUM series. Sequences with identities above a given identity cutoff are clustered and used to derive score matrices. The BLOSUM matrices are known as a good general-purpose choice. Especially, BLOSUM62 is frequently chosen for the alignment of sequences.

## Results

We here introduce *Scoredist*, a novel correction-based distance estimator for protein sequences. It applies a correction function to an observed reduction in normalised score, rather than to observed differences as other correction-based methods. This gives a better estimate of the divergence in the well-established PAM measure and allows the popular BLOSUM matrix series to be used. Other matrices could in principle be used, but the BLOSUM matrix has proved to be the most universal. *Scoredist *distance estimates are calculated directly by a simple equation and do not require cumbersome computational approximations, which is needed for *e.g. *Maximum Likelihood (ML) and Expected Distance (ED) estimates [15]. Additional calibration opens the possibility to make *Scoredist *tuned to other evolutionary models.

In order to evaluate our novel protein distance estimator *Scoredist *against other estimators, we generated a large testset of artificial sequence alignments. Simulation is the only way to exactly know an alignment's evolutionary distance. The substitutions were made by ROSE [16] according to an evolutionary model that can be chosen arbitrarily. It is to be expected that a distance estimator based on a particular evolutionary model will perform optimally on a testset generated with the same model. We therefore generated testsets using four different matrix series: Dayhoff [10], MV [12], JTT [11], and WAG [13]. For each model, 2000 alignments were created for evolutionary distances between 1 and 200 PAM units, i.e. 10 alignments for each distance. The *Scoredist*, Maximum Likelihood, and Expected Distance estimators can all be tuned towards a particular evolutionary model. We therefore used three evolutionary models which were also used to generate the testsets for these distance estimators, and use a shorthand to refer to these as "method-model". For instance, Maximum Likelihood using the MV model is denoted ML-MV. The Jukes-Cantor and Kimura estimators can not be tuned to a specific model but were tested on all four datasets.

Table 1 shows a compressed summary of the results. For each combination of distance estimator and dataset, the average root mean square deviation from the true distance was calculated for all 2000 alignments. The Expected Distance results were similar to ML, as the methods are akin in nature, but ML was generally more accurate and is much more widely used. As expected, low RMSD values as a sign of good distance estimates were generally obtained when using the same model for alignment creation and subsequent distance estimation. This is seen in the diagonal of low RMSD values from Dayhoff/Dayhoff to JTT/JTT. The only exception to this rule was observed when the testset was generated with Dayhoff. Here, ML-JTT was slightly better than ML-Dayhoff. This result was also verified for distances up to 250 and 300 PAM (data not shown). Comparing *Scoredist *and ML accuracies when training and testing using the same model resulted in a tie. *Scoredist *was better for MV, ML was better for JTT, and they were equally accurate for Dayhoff. When comparing accuracies for different training and testing models, however, *Scoredist *dominates. Here, *Scoredist *performed better than ML in five of the six cases. For the MV testset the difference was very big. The only case where ML was better than *Scoredist *was again when running ML-JTT on the Dayhoff testset, which for unclear reasons produced very accurate distance estimates.

The Jukes-Cantor and Kimura correction methods are generally less accurate than *Scoredist *and ML estimators. In some cases they reached higher accuracy than *Scoredist *and ML trained on the "wrong" model. For instance, on the Dayhoff testset Kimura was better than *Scoredist*-MV and ML-MV, and on the MV testset Jukes-Cantor was better than *Scoredist *and ML trained on Dayhoff or JTT. However, Jukes-Cantor and Kimura never came near the *Scoredist *and ML accuracy when trained on the "right" model. In a real situation, it is of course not known which evolutionary model is most appropriate. Therefore, taking the average RMSD values for each training model reveals the generality and robustness of the method on different testsets. The average accuracy of *Scoredist *is consistently better than for ML, and Jukes-Cantor and Kimura are even further behind.

**Figure 1:**
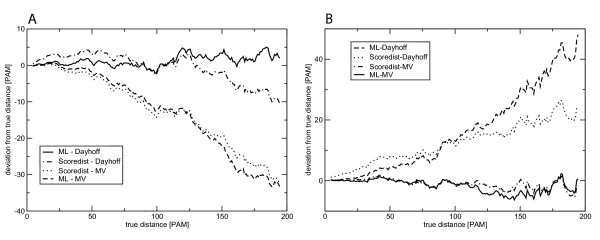
**Stratified accuracy analysis of *Scoredist *and ML**. To illustrate how estimated distance depends on the model, the average deviation is plotted as a function of true distance for two evolutionary models, Dayhoff and Mueller-Vingron. For each evolutionary distance between 1 and 200 PAM, 10 alignments were generated. For each alignment, the deviation was calculated as the difference between the estimated distance and the true distance used for data generation by ROSE [16]. The average of the 10 deviations was plotted using a running average with a window of 10 residues. Note that positive and negative deviations at the same true distance can cancel each other out – the curve only shows the average deviation and not the variability. The values in Table 1 measure the accuracy more correctly by using RMSD of every datapoint. The testset data was created with the matrices given by Dayhoff (A) or Müller-Vingron (B). In both cases, the estimators using the same evolutionary model as the testset data perform well. However, when switching the model in the estimator, *Scoredist *diverges less than ML, indicating that *Scoredist *is more robust. The curves show that ML-MV is more different from ML-Dayhoff than *Scoredist*-MV is from *Scoredist*-Dayhoff, particularly for the MV dataset in (B). The less difference between estimates using different models, the more robust is the method.

Figure 1 two shows a more detailed picture of the different distance estimators. The average of 10 estimates from 10 independent simulations at each evolutionary distance is plotted for data generated with the Dayhoff matrices. The variance among the 10 estimates is not shown for clarity; they are however reflected by the RMSD values in Table 1 which may give a slightly different picture. For instance, it is possible that the average deviation is close to zero if the individual estimates have large positive and negative deviations that cancel each other out. Therefore, the RMSD values should be trusted more than the deviation plots when in doubt. Figure 1A shows the dependence on evolutionary model for *Scoredist *and ML. Testing on the Dayhoff testset, *Scoredist*-Dayhoff and ML-Dayhoff stayed reasonable accurate in the entire range (below 5% error). In contrast, *Scoredist*-MV and ML-MV deviated considerably from the true distance. It is however clear that ML is more affected by switching model than *Scoredist *is. In Figure 1B the testset was generated with the MV model. Again, the corresponding deviation was observed for "wrong model" estimators. Here it is even more pronounced that ML is more dependent on the model, and generalizes poorly. *Scoredist *was less affected by the change of model – *Scoredist*-Dayhoff was considerably more accurate on the MV testset than ML-Dayhoff. As expected, when *Scoredist *and ML had been trained on MV data, the accuracy is very good for both estimators. In conclusion, we observed that although the *Scoredist *method is very simple compared to the ML method, it is approximately equally accurate when testing and training using the same evolutionary model. However, when testing on a different model, *Scoredist *is considerably more accurate.

## Implementation

The *Scoredist *estimator was implemented in Belvu, which is a general-purpose multiple alignment viewer that allows basic alignment editing. Belvu can calculate and display phylogenetic trees. The tree reconstruction can be based on *Scoredist *or other common correction-based distance estimators available within Belvu.

**Figure 2:**
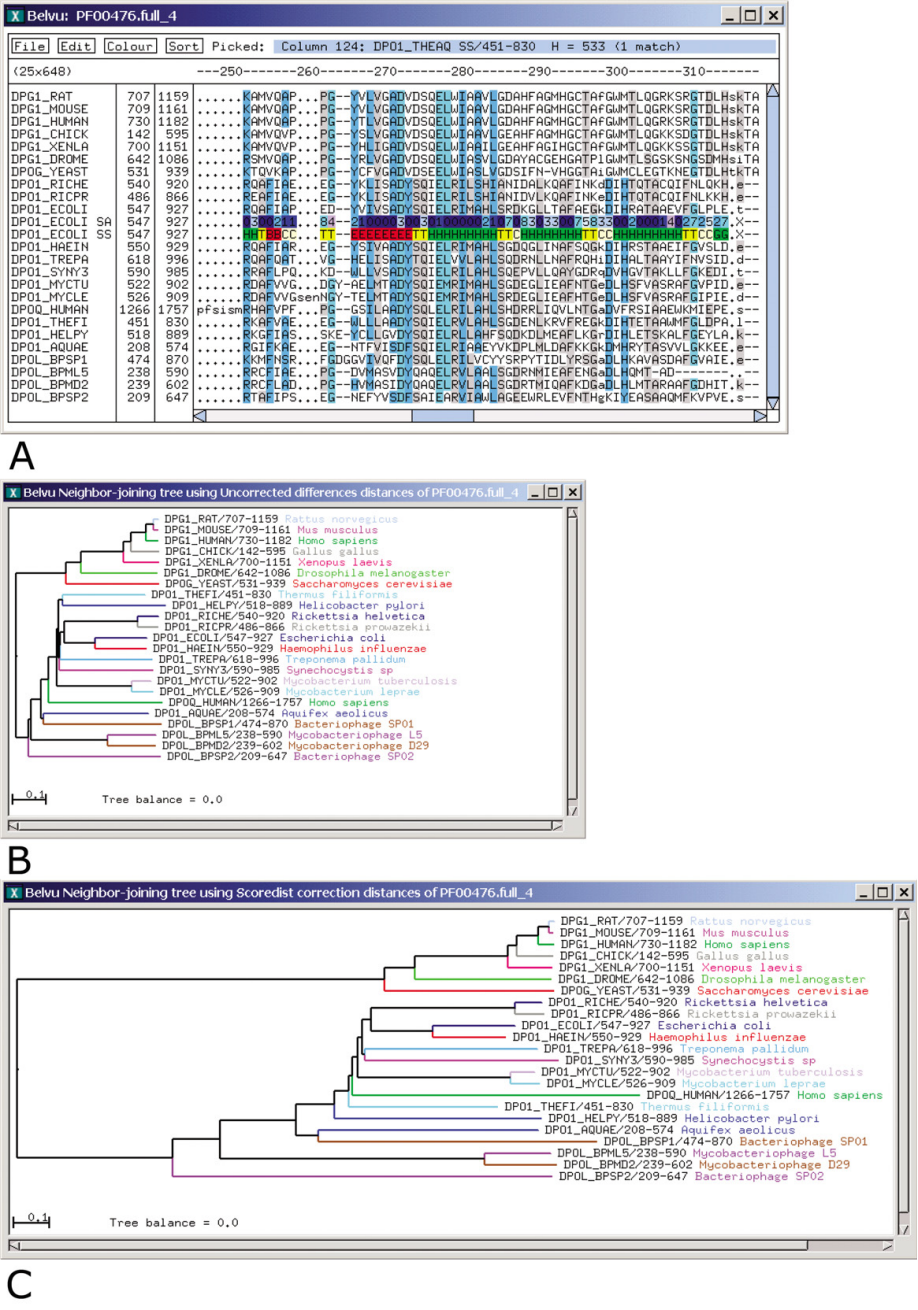
**The Belvu multiple sequence alignment viewer**. Belvu is a multiple sequence alignment viewer that implements the *Scoredist *distance estimator. The alignment window (A) shows a subset of the Pfam family DNA_pol_A (PF00476). Uniprot IDs are shown throughout. A sequence with known structure is included (DPO1_ECOLI) – the SA line showing surface accessibility and the SS line showing secondary structure. The neighbour-joining tree in (B) used uncorrected distances (observed differences), while the tree in (C) used *Scoredist *correction. Belvu assigns a colour to each species if provided with species markup information. The distance correction mainly affects the longer branches, and affects the tree topology in some cases, *e.g*. the placement of DPOQ_HUMAN. Structural markup and taxonomic information were embedded in the Stockholm format alignment provided by the Pfam database.

Multiple alignments can be coloured in Belvu according to conservation using average BLOSUM62 score in the column, or by residue-specific colours. User-specified cutoffs can be employed to fine-tune the display. Belvu has a range of functions for sorting, colouring, marking up, and printing alignments. In Figure 2, the alignment is coloured according to conservation, and sorted according to the tree. The effect of distance correction with *Scoredist *is illustrated.

Belvu can also be utilised for batch mode operations on the multiple alignment, or for producing distance matrices or phylogenetic trees without graphical output. It is available for the most common UNIX operating systems and can be obtained from [20]. A Windows version exists but is less frequently maintained. See [17] for instructions, and [18] for information on the Stockholm format, which is used by the Pfam project.

## Discussion

Our analysis was based on four different evolutionary models – Dayhoff, MV, JTT and WAG. We chose these because they represent the spectrum of models well. The only tuning done in the *Scoredist *method is the estimation of the calibration factor *c*. This factor can be seen as a scaling factor for the logarithm base in equation (5) that needs to be set empirically.

The difference between *Scoredist *and ML becomes particularly apparent in the MV dataset. There are several hypotheses for this behaviour. The Dayhoff matrices were constructed with the limited data available at the time. Given the substantial increase of research output in this field particularly during the last decade, it is not surprising that the Müller-Vingron model (published in 2000) reports substantially other results than the Dayhoff (1978) and JTT (1992) matrices. Additionally, the calibration factor *c *can also be interpreted as measure for the similarity of the respective models. Following this argument, JTT and Dayhoff are more akin given a Δ*c *≈ 0.05. The MV model is more distant to both JTT (Δ*c *≈ 0.11) and Dayhoff (Δ*c *≈ 0.16).

The Expected Distance estimator generally overestimates distances. For instance, among Dayhoff-calibrated estimators on the MV testset, Expected Distance is more than 10 PAM RMSD units (over 50%) poorer than the best method *Scoredist*. Similar values are observed for JTT calibrated estimators. Generally, MV-trained estimators are prone to underestimate evolutionary distances (Figure 1A). In combination with the ED higher distance estimation, this rather fortuitously leads to good results for ED – MV. However, the scope of this research was to identify a robust method that performs well on various data sources. An estimator which is highly sensitive to the data source or possible incorrect calibration is of less value. The best single estimator was JTT-calibrated *Scoredist*. If the method *per se *is measured by averaging over all calibrations and testsets, *Scoredist *receives 15.39, ED 16.89, and ML 17.14 PAM RMSD units. This highlights *Scoredist *as the most robust estimator, with the distance between *Scoredist *and ED (Δ_*Scoredist*, ED _≈ 1.50) being 6 fold the difference between ED and ML (Δ_ED, ML _≈ 0.25).

We here only present *Scoredist *results using BLOSUM62 for calculating the score *σ *between two sequences. In principle one could use some other score matrix, but we found that this had little effect on the results. Since the goal was to make a general-purpose method, BLOSUM62 was an obvious choice. The key to *Scoredist *is the usage of scores rather than identities, and the choice of somewhat arbitrary parameters is not of primary concern. At present, gaps in the alignments are not included in the *Scoredist *calculation. Traditionally, gaps have been difficult to embody in evolutionary models. In the models used here, they are at best crudely modelled by treating every gap equally. An inherent problem is that the probabilities for insertions and deletions (indels) are not necessarily synchronized with the substitution probabilities. Some protein families are more prone to indels than others, hence it is hard to make a generalizable model that suits all protein types. We have experimented with affine gap penalties in the *Scoredist *method (this is an option in the implementation), but this resulted in decreased accuracy. We therefore do not recommend using gaps to estimate protein distances.

## Conclusion

We have developed the score matrix based distance estimator *Scoredist *for aligned protein sequences. Its main advantages are computational simplicity and high robustness. Most other distance estimators produce good results for certain evolutionary models but perform poorly on others. The Maximum Likelihood and Expected Distance were found to overfit their estimates to the evolutionary model so much that the results on testsets generated with other models suffered heavily. The correction-based methods Jukes-Cantor and Kimura also favoured a particular evolutionary model, but were not competitively accurate on any testset. It seems that *Scoredist *achieved the best compromise between accuracy and generalization power.

## Methods

For the estimation of divergence time, let s^1 ^and s^2 ^be two aligned protein sequences (gaps are ignored) of identical length *l*. A similarity score *σ *is defined as



where *S *is a log-odds score matrix. Log-odds score matrices are constructed such that substitutions by the same or a similar amino acid receive a positive score, whereas substitutions to dissimilar amino acids are attributed a negative score. The expected value for this kind of matrix is negative. This ensures that the comparison of unrelated sequences returns a negative score. For two random sequences of length *l *the expected score *σ*^*r*^(*l*) = *σ*^0 ^* *l*, where is the expected value of the score matrix. As we strive to measure scores above the scores for the null model of sequence independence, the score *σ*(*s*^1^, *s*^2^) is deducted by the expected score *σ*^*r*^, giving the normalised score *σ*^*N*^

*σ*^*N *^= *σ*(*s*^1^, *s*^2^) - *σ*^*r *^(*l*).     (2)

For two random sequences of length *l *the expected score *σ^r ^*(*l*) = *σ*^0 ^* *l*, where *σ*^0 ^is the expected value of the score matrix.

The expected score *σ*^*r *^for unrelated sequences can be regarded as lower limit. The upper limit of the score between *s*^1 ^and any other sequences is given by *σ*(*s*^1^, *s*^1^). For two different sequences, the upper limit of the score *σ*^*U *^is, for the sake of symmetry, assumed to be



and normalised

*σ*^*UN *^= *σ*^*U *^(*s*^1^, *s*^2^) - *σ*^*r *^(*l*).     (4)

Any sound score *σ^N ^*is situated within the interval [0, *σ^UN^*]. The validity of the upper boundary follows from the score's definition. The lower boundary might, however, get violated if two sequences receive a score *σ*(*s*^1^, *s*^2^) <*σ^r ^*(*l*). As the model assumes independent evolution already for *σ*^*r *^(*l*), a score below *σ*^*r *^does not contain any additional information. A lower score is therefore set to *σ*(*s*^1^, *s*^2^) = *σ*^*r *^(*l*). We model the raw distance as a modified Poisson process



**Figure 3:**
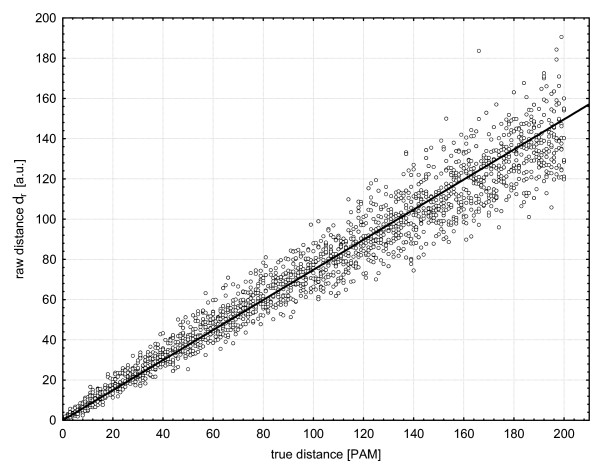
**Estimation of the calibration factor *c *in *Scoredist***. This factor rescales the raw distance *d*_*r *_to optimally fit true evolutionary distances. The plot shows how *c *is estimated by least-squares fitting of raw distances *d*_*r *_to true distances for 2000 artificially produced sequence alignments, using the Dayhoff matrix series. The linear relationship between the raw distance *d*_*r *_and the true distance of the sequence samples justifies the introduction of the calibration factor *c*, which was here determined to *c*_*Dayhoff *_= 1.3370 (See Table 2).

As seen in Figure 3, *d*_*r *_is linearly related to the true distance, deviating only by a constant factor. The *Scoredist *evolutionary distance estimate of two sequences is given as the product of the raw distance and a calibration factor

*d*_*s *_= c * *d*_*r*_.     (6)

Evolutionary distances of 250–300 PAM units are commonly considered as the maximum for reasonable distance estimation and, therefore, the *Scoredist *estimate *d*_*s *_is restricted to the interval [0, 300] PAM.

Calibration factors can be determined for various evolutionary models. We used the ROSE program [16] to simulate evolution with three different matrix series and generated 2000 sample sequence alignments for distances up to 200 PAM units. The calibration factor *c *was calculated by least squares fitting on this data, using the BLOSUM62 score matrix for calculating the score *σ *in the estimator (Table 2, Figure 3). The simulated evolution started with a random sequence of 200 residues. For each integer distance within the interval [1, 200] PAM, we produced 10 alignments, yielding 2000 alignments per dataset. The default gap parameters of ROSE V1.3 were applied. Each dataset was generated with the transition probability matrix and the stationary frequencies of the respective evolutionary model.

Calculation of Maximum Likelihood (ML) and Expected Distances (ED): ML distances were estimated by applying the Newton-Raphson method to the derivative of the likelihood of the evolutionary distance given an alignment. To calculate ED, the same likelihood function was numerically integrated, to get its "center of gravity" [15]. Both methods are implemented in the program lapd (L. Arvestad, unpublished), which uses Perl and Octave. The Jukes-Cantor and Kimura distance estimators were run as implemented in Belvu. The popular PROTDIST program from the PHYLIP package [19] calculates only ML-Dayhoff and Kimura distances. We therefore chose to use lapd in order to assess *Scoredist *by a broader range of distance estimators.

## Authors' contributions

ES had the initial idea and implemented the method. VH carried out the evaluation and wrote the first manuscript draft. All authors read and approved the final manuscript.

## Figures and Tables

**Table 1:** Accuracy as average RMSD values for combinations of data modelsand estimators

	testset				
		
	Dayhoff	MV	JTT	WAG	average
Scoredist – Dayhoff	12.68	20.85	13.67	12.81	15.00
ML – Dayhoff	12.70	28.40	14.75	15.15	17.75
ED – Dayhoff	13.57	31.36	16.10	16.63	19.41
Scoredist – MV	19.28	13.15	16.29	18.73	16.86
ML – MV	19.96	13.44	19.36	19.21	17.99
ED – MV	15.68	13.35	13.95	14.75	14.43
Scoredist – JTT	13.67	17.16	12.89	13.47	14.30
ML – JTT	12.15	25.07	12.10	13.44	15.69
ED – JTT	12.56	27.71	12.70	14.37	16.84
Jukes-Cantor	23.92	16.28	19.88	22.48	20.64
Kimura	16.24	29.81	22.36	19.16	21.89

For each testset and method, the average root mean square deviation from the true distance was calculated for 2,000 alignment samples in the interval 1–200 PAM units. Lower RMSD values indicate higher accuracy on a single testset. The column 'average' gives the mean of the four evaluated testsets. A low value in this column shows the estimator's robustness as it measures the accuracy over all four models (including "wrong" data models). *Scoredist *was more robust than ML, as it for each training set always had higher accuracy on average. The ED estimator gave good results when trained with MV, but was poor in all other cases (see Discussion for details). *Scoredist*, Jukes-Cantor, and Kimura distances were calculated with the Belvu alignment viewer. The Maximum Likelihood (ML) and Expected Distance (ED) estimates were produced by lapd (L. Arvestad, unpublished).

**Table 2:** Calibration factors for three evolutionary models

	*c*
Dayhoff	1.3370
JTT	1.2873
MV	1.1775

The raw distance *d*_*r *_is scaled by the calibration factor *c*, which was obtained by least squares fitting of 2000 artificial protein sequence alignments generated for the matrices as given by Dayhoff, JTT (Jones-Taylor-Thornton), and MV (Müller-Vingron).
